# Injury Characteristics and Physical Therapy Management Strategies for Circus Artists: A Scoping Review

**DOI:** 10.3390/jcm14175948

**Published:** 2025-08-22

**Authors:** Jessica Straub, Dhinu J. Jayaseelan, Clara Honigberg, David A. Scalzitti

**Affiliations:** 1 Alliance Physical Therapy, Baltimore, MD 21211, USA; 2 Program in Physical Therapy, The George Washington University, Washington, DC 20006, USAscalzitt@gwu.edu (D.A.S.)

**Keywords:** performing arts, physical therapy, rehabilitation

## Abstract

Background: Circus arts are gaining popularity across the globe and lifespan. Despite growing participation rates, there is limited high-quality evidence describing the physical therapy evaluation and management of injury within this population. The purpose of this scoping review was to identify and summarize evidence related to the injury characteristics and management of circus artists. Methods: With a research librarian, computerized searches were developed and performed using online databases (PubMed, CINAHL, Scopus, SPORTDiscus and Cochrane), gray literature and non-medical databases. Two authors independently voted on article inclusion with a third author blinded to previous voting used in case of conflict. Concept mapping identified five primary themes related to circus injuries and physical therapy treatment. Data was extracted from each article based on content relevant to the classification. Results: In total, 1095 titles were retrieved from electronic searches, of which 49 studies were included. Overall, 20 studies related to injury characteristics, 10 to risk factors, 9 to each psychosocial variables and interventions, and 8 to screening. Acrobats with required ground elements were the most commonly injured discipline, particularly at the shoulder, ankle and spine. Intrinsic and extrinsic risk factors were identified and numerous psychosocial and lifestyle contributing factors were reported. Literature guiding treatment is limited. Conclusions: This scoping review mapped and synthesized evidence related to the evaluation and management of circus artist injuries. While injury patterns and characteristics are generally understood, the treatment of this population is poorly described. Clear gaps in screening, injury prevention strategies, and interventions for this group were identified.

## 1. Introduction

Circus arts are practiced globally in a variety of contexts, with expertise ranging from professional companies to recreational participants. In the United States participation in the circus arts has grown substantially, with more than 78% of the circus organizations founded after 2000 [1]. Of these circus organizations, 74.4% offer adult recreational classes. The circus arts are highly demanding athletic activities that require similar strength, balance, flexibility, and motor coordination to gymnastics [2], and activity that carries substantial injury risk [3]. Coupled with these sport-specific demands, there is often a high work load and short recovery time due to frequent performance schedules [4]. Even for professionals, performance and training may require considerable travel, and access to appropriate care may not be perceived to be available [5]. Respecting these variables, it becomes apparent that circus artist athletes are unique and their sport complex, and physical therapy management requires a deep understanding of the population.

Unfortunately, despite the need for high-quality evidence guiding management, available evidence includes inconsistent injury definitions for circus arts, making it difficult to compare and contrast trends. In 2020 Greenspan et al. [6] created an International Olympic Committee (IOC) consensus statement on reporting injury. The consensus statement comes after a 2017 systematic review including literature through March 2016, reporting substantial variance and a need for consistent reporting of musculoskeletal circus injuries [7]. The review noted a relatively low injury rate among professionals, ranging from 7.37 to 9.27/1000 artist exposures, mostly affecting the spine and ankle. Although not captured in the previous review, it should also be noted that circus artists may suffer from substantial emotional distress [8] which may elevate their risk of injury [9]. In addition to the substantial psychosocial demands, evaluation and management of injury is likely made challenging by the wide-ranging task-specific demands of circus arts. Greenspan [10] separated ground and aerial activity due to the differences in physical demands. Subgroups consisted of aerial acrobatics with ground elements, aerial acrobatics, ground acrobatics with human propulsion, ground acrobatics with balance/control, manipulation, and character. More research is needed to precisely describe injury patterns of this specialized group of athletes.

In addition to understanding injury patterns among circus artists, it seems appropriate to investigate factors that may elevate risk and develop instruments to identify potential risk prior to injury. Without validated pre-participation screening instruments, tools used in other similar sports (e.g., gymnastics) could be useful. In fact, perhaps due to sport-specific similarities, some gymnasts transition to careers in circus arts [11]. However, while the evaluation and management of gymnastics injuries has been well described [12,13,14,15], the management of circus artist injuries is not. In fact, there has not been a single randomized controlled trial with circus artist participants. Clinical reasoning and treatment decision making should be guided by high-quality evidence, but none appears available for circus artists. In order to create baseline knowledge to guide clinicians, an extensive evaluation of available evidence describing the treatment of the injured circus artist is required.

Despite the rapid increase in circus arts participation at the professional and recreational level and subsequent injury risk, there is limited evidence describing circus arts, particularly the contemporary injury patterns and treatment approaches. Synthesized evidence can help to inform clinical practice and identify literature gaps to guide future research, but such evidence does not exist for this unique population. This scoping review aims to identify and report on the injury characteristics and management strategies for circus artists.

## 2. Materials and Methods

This scoping review was pre-registered on the Open Science Framework (OSF) website 8 March 2024, and is freely available online. Reporting followed guidance of the Preferred Reporting Items for Systematic Reviews and Meta-Analyses extension for scoping reviews (PRISMA-ScR).

### 2.1. Data Sources and Searches

A comprehensive and systematic computerized search of the electronic databases PubMed, Scopus, CINAHL, the Cochrane library, and SPORTDiscus was conducted to identify articles relevant to the clinical question. All articles from database inception through 3 September 2024 were considered for inclusion. With the assistance of a research librarian, key search terms and various combinations of synonyms related to the concepts of injury characteristics and management of circus artists were entered into each database. Specific search strategies are presented in the Supplementary Materials. The reference lists of eligible articles were manually examined for key review articles and additional relevant studies. Finally, clinical trial registries, Google, and the Open Grey database were scanned for pertinent work not captured otherwise.

A basic example search string is as follows, with relevant truncation, adjustments, Boolean operators, and MeSH terms used depending on the database: (“circus artist*” OR circus OR aerialist* OR acrobat*) AND ((“Wounds and Injuries” [Mesh] OR “Epidemiologic Methods” [Mesh]) OR (injury AND (prevention OR assessment OR screening)) OR (“Psychiatry and Psychology Category” [Mesh] OR psychosocial) AND (“Physical Therapy Modalities” [Mesh] OR “physical therapy” OR physiotherapy OR rehabilitation OR recovery OR “conservative management”)).

### 2.2. Eligibility Criteria

**Figure 1:**
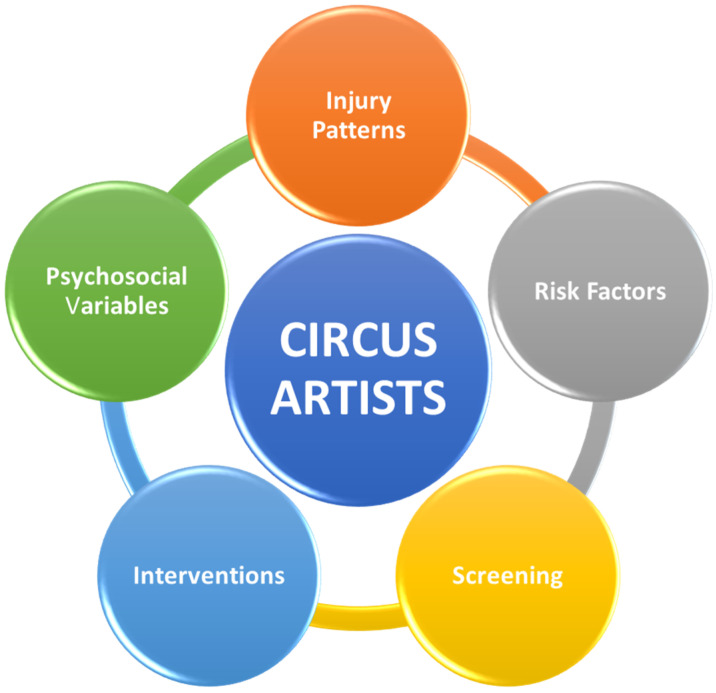
Concept map of circus artist injury characteristics.

Studies were considered in this review if they related to the clinical question and had a full-text report published in the English language in a peer-reviewed journal. The clinical question was categorized into thematic areas after concept mapping was completed by the research team (Figure 1). For inclusion, the population of interest was circus artists and the study aim was related to any of the following: injury trends or characteristics, risk factors, screening or injury prevention instruments, physical therapy management or psychosocial variables. Studies were excluded if they were published in abstract form only or protocols, were not in the English language or reasonably translated to English, not related to circus artists, primarily related to gymnasts or dancers, or if they were not within the scope of injury characteristics or management.

### 2.3. Study Selection

Prior to screening, all individuals involved in screening used 10 titles for a reliability exercise. Each individual independently voted for inclusion or exclusion, and upon completion, the authors met to discuss results, appropriateness for decision making, and create a consensus for future screening principles. After the electronic search was completed and duplicates removed, two reviewers independently screened the titles and abstracts for eligibility using the web-based literature review manager Covidence, with criteria determined a priori. After the preliminary search of the above databases, any article that included circus artists and injury characteristics or management was retained for further analysis. In cases where details of the study methods were unclear, the study’s corresponding author was contacted for additional information. Amongst those articles in which disagreement occurred, a third author blinded to previous voting made the final decision for inclusion. Full-text articles were procured through institutional access, interlibrary loan, or requesting copies from the corresponding authors. After full-text articles were obtained, two reviewers independently evaluated the study for appropriateness. If consensus regarding inclusion was not achieved, a third author blinded to previous voting was consulted for a final vote to include or exclude the article.

### 2.4. Data Extraction and Reporting

Upon final full-text article inclusion, data from studies were extracted to standardized forms. Grouping of data was subclassified into the 5 thematic areas. In cases where studies were pertinent to multiple concepts (e.g., injury patterns and risk factors), data was extracted and reported for each rather than one. Data was extracted by an author and independently cross-checked for accuracy and consistency by a separate author. An assessment of available evidence, and strength of evidence, was synthesized qualitatively.

### 2.5. Quality Assessment

The purpose of this scoping review was not to determine individual study quality as much as the overall quality of evidence related to a given topic. No formal assessment of study quality was performed for this project.

## 3. Results

**Figure 2:**
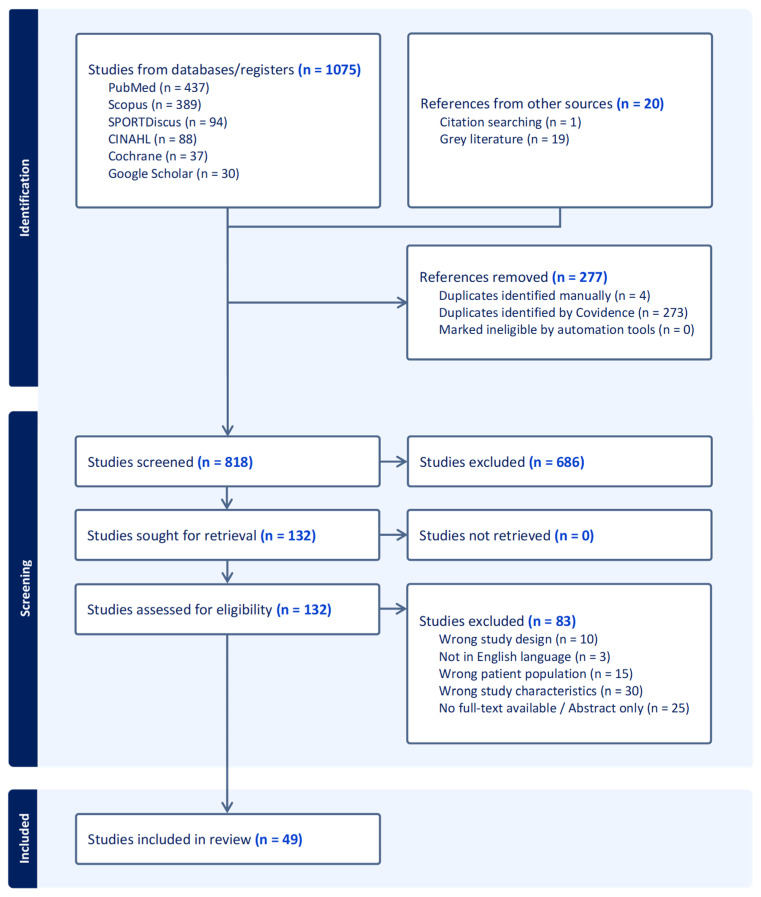
PRISMA flow diagram.

**Figure 3:**
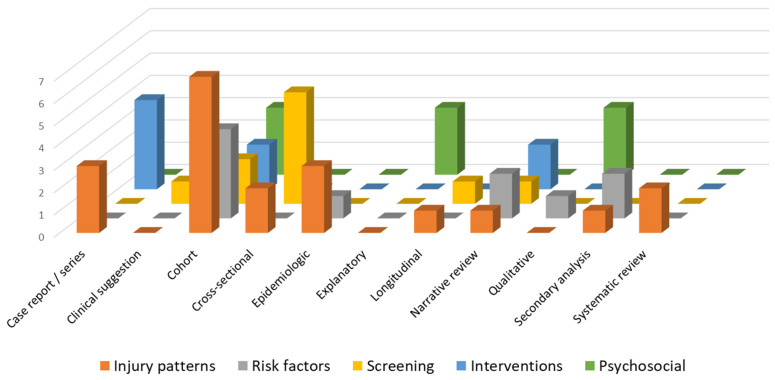
Study design type frequency by characteristic.

**Figure 4:**
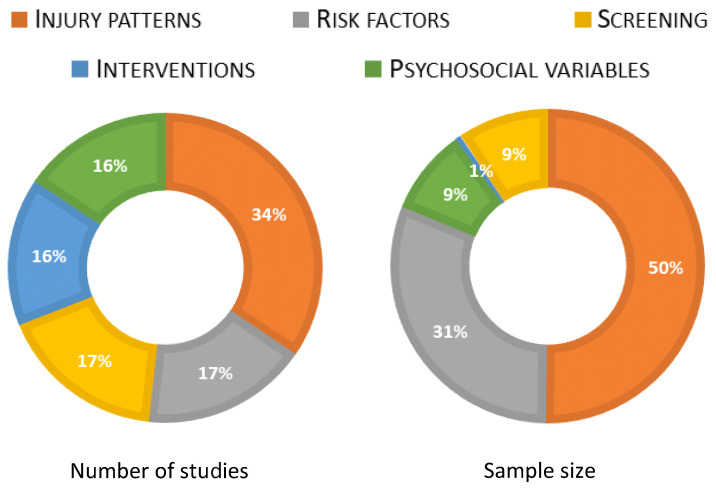
Proportion of included studies (**left**) and sample size (**right**) by injury characteristic.

In total 1094 titles were retrieved from the searches. After the removal of duplicates, 817 studies were screened, of which 49 studies were retained for final inclusion in this scoping review (Figure 2). The study design and primary themes of the studies are presented in Figure 3. The highest proportion of studies were related to injury characteristics (n = 20) followed almost equivalently by studies related to interventions, psychosocial variables, risk factors and screening methods for circus artists. Nine studies were tagged with multiple classifications. Less consistent than the number of studies evaluating a given topic was the sample size associated with the topic. Of the 10,272 unique participants included in all retained studies related to circus artists, 5397 (50.26%) participants were included in the 20 studies examining injury characteristics as compared to only 57 (0.55%) participants in the 9 studies describing management of injury (Figure 4).

### 3.1. Injury Patterns

The type of studies related to injury patterns is presented in Figure 3. Of the 20 studies, there were 2 systematic reviews [7,16], 7 cohort studies [2,4,10,17,18,19,20], 1 longitudinal study [21], 2 cross-sectional studies [22,23], 2 descriptive epidemiological studies [24,25], 1 retrospective descriptive epidemiology study [26], 1 case series [27], 1 narrative review [28], and 2 case reports [29,30]. Sample size ranged from 1 to 1376 participants with a diverse level of performer expertise (e.g., professional versus pre-professional, adolescents versus adult circus performers). There was notable variation in injury reporting, particularly as it related to the athlete characteristics, type and impact of injuries. Specific data from individual studies is presented in Table 1.

Of the 20 studies, 9 did not specifically report the mechanism of injury. There were five studies reporting overuse conditions to be most prominent, five reporting traumatic injuries to be most prominent, and one study noting that trauma occurred more frequently in adolescents while overuse was more common in adults. One study used the recently developed circus-specific extension of the International Olympic Committee injury recording guidelines [10], while two studies used and adapted version of the International Association of Dance Medicine and Science (IADMS) Standard Consensus Initiative guidelines [2,17]. Outside of these studies, reporting differed across author groups.

Grouping of participant discipline for injuries varied between studies. Greenspan [2] described circus athletes in the following groups: aerial acrobatics with ground elements, aerial acrobatics, ground acrobatics with human propulsion, ground acrobatics with balance/control, manipulation, and character. A separate study used acrobat, non-acrobat, and musician to broadly classify circus artists [24]. The authors used the terms acrobat to describe a performer that required gymnastics, diving, martial arts, and aerial movements, non-acrobat to describe dancers, jugglers, swimmers, clowns, and characters in a show that do not perform gymnastic or martial arts or aerial movements, and musician to describe people that play music. Alternatively, Orlando et al. [20] utilized the terms sudden load, non-sudden load, and musicians to categorize circus injury.

**Figure 5:**
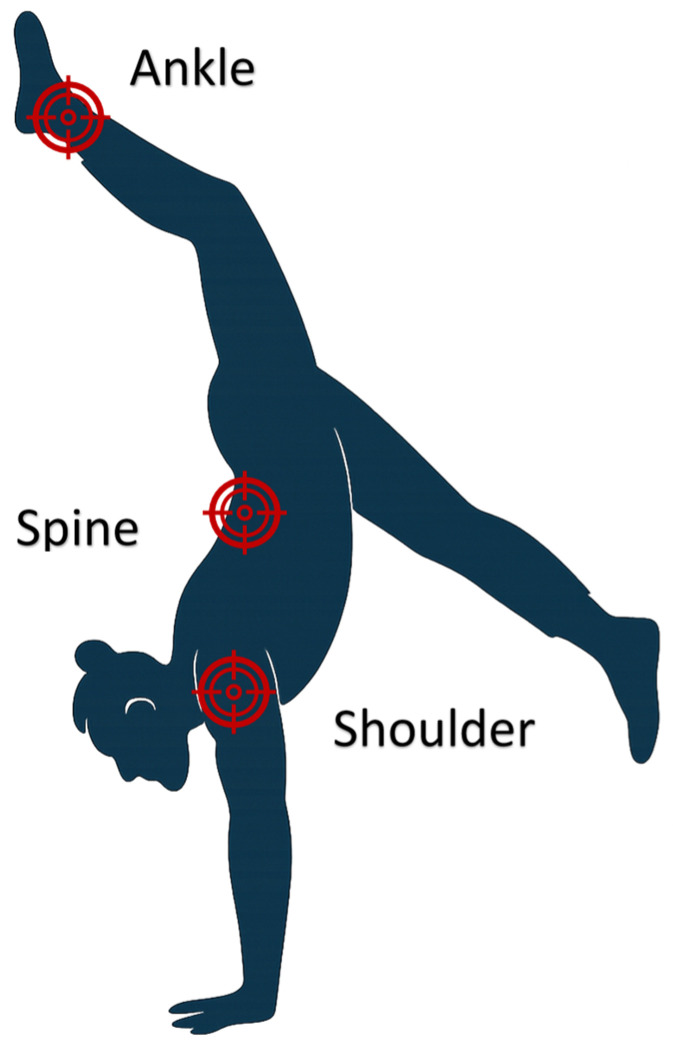
Most frequently reported circus injury sites.

The types of injuries encountered were more consistent across studies than the way injuries were reported. Injury rates ranged from 1.89 to 9.7; however, studies varied rates based on hours or exposures. Acrobats who performed ground elements were the most commonly injured discipline. Although most injuries likely led to some time away from their sport, time lost (TL) injuries were explicitly detailed in 7/20 (35%) studies. When considering the type of tissue injured, three studies reported joint injuries to be most common while three studies noted muscle/tendon injuries to be most common. While all body regions were prone to injury, the most commonly injured sites are shown in Figure 5.

### 3.2. Risk Factors

Of the 49 studies included, 10 studies [9,10,17,20,25,28,31,32,33,34] were related to risk factors (Figure 3) with a total of 4459 participants analyzed. Extracted data from individual studies is presented in Table 2.

Risk factors were analyzed in extrinsic and intrinsic categories. Extrinsic risk factors were broadly related to performance demands and environment (high performance workload and frequency with a lack of rest [20,32,33]). Pre-professional artists had higher injury rates (4.08/1000 sessions) compared to professional artists (3.49/1000) and those requiring sudden-load discipline demands also were at higher risk than other disciplines (5.93/1000 to 4.26/1000) [10,20]. Qualitative data identified poor touring conditions and weather as a risk factor for injury [32]. Intrinsic risk factors included older age [10,17,34], female sex [10,34], disordered eating [10], and psychological factors (e.g., self-efficacy, emotional exhaustion, higher mental load) [9,32,33]. There were conflicting results related to previous injury being a risk factor for a future injury [9,20,31], which is consistent with Shrier et al. [25], who reported that observed injury risk was not consistent with theoretical injury risk.

### 3.3. Screening

Of the 49 studies included, 10 studies [23,35,36,37,38,39,40,41,42,43] were related to screening tools or principles in circus artists (Figure 3) reporting on a total of 913 participants, with individual study data presented in Table 3. Studies included participants across the expertise spectrum, from professionals with Cirque du Soleil through pre-professionals/amateurs, although participant skill was unspecified in three studies. Two screening-related studies were narrative reviews which suggested screening for physical performance, hypermobility, training and lifestyle [36] or using a movement system impairment classification [41]. Four of the studies [35,39,40,43] completed physical performance testing relevant to participant screening. Circus artists were found to have generally more than normal mobility although findings were not discipline-specific. Not all physical testing may provide consistent results; specifically, the Harvard step test, 60 s jump test, and dynamic balance test did not have good reliability in circus athlete testing [35]. Four studies [23,37,38,42] screened mental health, mood, lifestyle and perceptions of health. Studies found self-reported outcomes were feasibly completed and students had different baseline measures compared to professionals. Instruments identified different mood levels at different time points (e.g., beginning versus end of a semester) although surveys can be perceived as burdensome if distributed too frequently [42].

### 3.4. Interventions

Of the 49 studies included, 9 were related to the physical therapy management of circus artists (Figure 3) with a total of 57 participants included [33,36,41,44,45,46,47,48,49]. Intervention-specific data is presented in Table 4. Narrative review and suggestion topics were related to making interventions patient-specific, using a movement systems-based approach, and using an interdisciplinary management strategy. Cohort design studies reported on the use of telehealth to treat atraumatic shoulder instability or feasibility of a calf muscle strengthening program. Case reports detailed the management of traumatic soft tissue tears or symptoms associated with hypermobility. No randomized controlled trials or comparative studies examining the effects of interventions for the circus artist were found in this scoping review. Of the five studies including the management of patients, as compared to suggestions, reviews, or feasibility studies, each reported clinically significant improvement in pain and/or functional outcomes. However, only three of the five studies (26 patients) reported on long-term follow-up [47,48,49].

### 3.5. Psychosocial Variables

Of the 49 studies included, 9 studies were related to psychosocial variables (Figure 3) [5,8,9,11,50,51,52,53,54]. A total of 961 unique circus artists were included with relevant study results presented in Table 5. There was a nearly equal range of expertise, as three studies included professional or retired circus artists [5,9,11], three studies included pre-professional circus artists [50,51,54], and three studies included professional and pre-professional circus artists [8,52,53]. Eight of the studies reported on participants’ mental health or emotional perceptions of their body, career, or lifestyle. Four of these studies reported on stress and negative emotions, two studies reported on fatigue, three studies reported on anxiety and fear, one study reported on depression, and one study reported on overall mental health. Overall, results indicated high levels of fatigue, negative emotions, increased anxiety, and low self-efficacy.

## 4. Discussion

This scoping review sought to describe injury characteristics and management strategies for circus artists. Circus arts participation is becoming more popular across the lifespan, and research is helping to identify injury trends and those at risk for injury. However, based on the results of this review, there appears to be substantial variation in reporting, few appropriate screening instruments for injury risk mitigation, and minimal evidence guiding the treatment of circus artist injuries.

Based on the results of this scoping review, the largest proportion of retained articles were related to the injury patterns and trends of circus artists. In a 2017 systematic review of circus artist injuries, Wolfenden and Angioi identified eight studies reporting on 4795 participants (1281 of which were duplicated from two studies) [7]. This scoping review found 11 new studies on injury patterns since that publication, suggesting a growing scholarly interest in the topic. Despite reporting variability, this review identified acrobats performing ground elements to be the most commonly injured discipline, the shoulder, ankle and spine to be common regions of injury, joint and muscle/tendon tissues to be commonly injured, and traumatic and overuse injuries to be equally common. Physical therapists are well-positioned to manage the gamut of circus injuries, but knowing the task-specific demands and mechanism of injury can allow for comprehensive and individualized care.

Variability and inconsistency across studies can make synthesis of findings difficult. Although a circus-specific reporting extension of the IOC consensus statement was published in 2022 [6], knowledge translation can take time, and only one study implemented the reporting methodology [10]. The lack of standardized surveillance, in part due to heterogeneity among study design and injury definitions, should be rectified for future prospective protocols across circus disciplines. Additionally, the potential for underreporting should be considered. In a recent systematic review, between 20 and 91% of workers failed to report their injury to their supervisors or worker’s compensation, with a variety of socioeconomic and employment characteristics, psychosocial variables, and healthcare providers often cited as contributing factors [55]. Circus artists may have pessimistic views of healthcare for their conditions and substantial internal and external pressure to push through injuries [5]. The quantitative results of this review related to injury patterns should be considered in context of possible underestimation.

This review identified intrinsic (personal) and extrinsic (environmental) risk factors that impact injury. Intrinsic risk factors identified were chronological age, history of eating disorder, and sex at birth, while extrinsic risk factors were type of performer, level of performer, and rest time between performances [17]. Greenspan [10,17] and Hamilton [34] found chronological age and age over 30 were associated with increased risk of injury. This contrasts with injury trends in gymnastics, where adolescents face a higher risk. The discrepancy may stem from differences in participation patterns, as adults are more likely to engage in circus arts, whereas gymnastics is predominantly practiced by younger individuals [56]. Individuals with an eating disorder averaged increased injury rates than individuals without an eating disorder [10], which is consistent with previous work in high school athletes [57]. This may be related to body image and esthetic expectations of circus performers [54] and possible exercise addiction [53] with implications for injury for sudden-load disciplines. Three of the studies noted discipline type as a risk factor. Greenspan [17] identified aerial with ground elements to have higher instances of injury per 1000 exposures, whereas Hamilton identified sudden load to have higher rate of time loss injuries [34]. Although higher workload and less rest between practice or performance were identified as risk factors for circus injuries [32,33], so too was being a pre-professional or less experienced artist [10]. Given the highly specialized and intricate physical demands of circus artists, good technique and appropriate rest are important factors in reducing injury risk. If clinicians are aware of common risk factors for injury in circus artists, they can create and implement screening and prevention strategies and create physical therapy plans of care that minimize risk of reinjury.

This review identified various screening tools and processes utilized for circus artists. Professional circus artists made up 68% of the studies included and pre-professional circus artists made up 32% of the studies. There was variation in assessment timing, which was collected at a single time point [35] or serially [23] at different points during a circus artists’ career. Callahan et al. [36] emphasized the importance of posture and movement retraining, education on when to use a mid versus full range of motion, and strength training to manage hypermobility. Although the authors did not suggest a specific strength training program, high-load strength training was superior in the short term to low-load training for individuals with hypermobile shoulders [58]. Long-term effectiveness was not different and the study did not include elite-level athletes, which limits generalizability to this population. Various testing modes including functional tests, manual muscle tests, dynamometers, and isokinetic testing were used to assess muscle performance [36,39,40,43]. The authors found that female circus artists tend to have greater passive range of motion compared to males, and professionals had greater muscle performance compared to pre-professionals. This may be a contributing factor associated with the greater injury risk of female athletes and pre-professionals.

Despite the substantial amount of evidence detailing injury patterns in the circus athlete population, there is a relative dearth of literature guiding the physical therapy management of this unique group. While some task-specific movements mimic those required in other well-researched sports (e.g., gymnastics, dance, cheerleading), the management of this group compared to its similar activities is nearly non-existent. The treatment of only 57 unique participants was identified with this review, approximately 0.5% of the total population assessed. Available evidence is limited to small cohorts, case studies, or suggestions, preventing the assessment of treatment effectiveness. From the limited evidence, clinicians can imply that circus artists who have substantial and unique physical and psychological demands require multimodal and tailored rehabilitation programs. One could postulate that the limited data describing circus athlete management parallels the psychosocial findings of this review, identifying a lack of willingness of circus artists to report injuries or perceived challenges with obtaining appropriate sport-specific care [5]. Recognizing that circus artists may be required to travel for performances, it may be difficult to establish continuous care. While telehealth options may be appropriate [46,47], classic in-person physical therapy care may be challenging and should be reconceptualized for this population. The discrepancy between rising circus performance popularity and injury research compared to intervention studies highlights the need for additional high-quality research.

This review found that circus artists and circus students reported high rates of mental health issues, including depression, anxiety, sleep deprivation, and stress. While none of the psychological studies included a control group of non-circus participants, 88.8% of the studies on psychological variables found emergence of negative emotions surrounding their craft [5,8,9,50,51,52,53,54]. Circus artists’ livelihood is dependent on how they are able to physically perform both during training and performances. Shrier [9] reported that high fatigue, emotional exhaustion, injury, and low self-efficacy put an individual at a 2–3× increased risk for injury. High stress and/or anxiety was reported in 55.5% of the psychological studies for circus artists [8,9,11,50,51]. Being impeded from doing one’s job can result in a mass of negative emotions, whereas having a more positive disposition has been found to improve long-term recovery and survival [59]. While circus artists experience many negative emotions, they also report a high amount of resiliency and motivation in their craft [8,11]. Poor sleep quality and high fatigue levels were another major theme reported among circus artists [9,50,51]. Previous research has suggested that a lack of sleep may put an individual at a greater risk of injury [60]. If an artist is stressed or anxious, this may lead to poor sleep, which in turn may predispose them to a higher likelihood of injury, which will begin a perpetuating cycle. This cycle has been previously discussed in the context of elite athletes [61]. Poor sleep hygiene also imposes a delay in injury recovery [62,63]. While impaired sleep and anxiety were not compared to one another in the included studies, these characteristics were reported in as high in 100% of the population in studies that reported data on those variables [9,37,51]. Evaluating negative psychosocial and lifestyle variables involved in an individual’s healthcare experience is essential, and physical therapists should recognize the prevalence of these problems in circus athletes.

While this review is novel and carries important clinical and research implications, there are limitations. The literature search included numerous databases and gray literature, and was created collaboratively by authors with systematic review experience and a research librarian. However, it is possible that potentially relevant work was not retained by the search itself or during the screening process. Specific clinical guidance offered from this report is limited, in large part due to limited evidence and variable reporting. However, the synthesis of key concepts related to the management of circus artists identified important information and opportunities for investigation. While not required for scoping reviews, if quality assessment was formally completed for the included studies, additional information could be provided regarding the topic.

While this review identified and synthesized key aspects of injury patterns and management strategies, a number of evidence gaps were recognized. The average age range across studies varied from 11 to 34 years old. According to the 2022 Circus Census Report, circus performers ranged in age from 6 to 55 and beyond [1]. Additional data on participants aged 6–11 and adults over 34 would help create a more comprehensive understanding of the entire circus artist community. Although the synthesized results of this review help understand injury patterns, it is clear that screening tools and management strategies are limited. Developing and validating sport- and discipline-specific physical and holistic screening instruments can allow clinicians to identify pre-participation injury risk and implement preventative programs to minimize or mitigate the risk. Finally, it is clear that although much is known about how circus artists get injured, and what their injury experience entails, the management of circus injuries is absent. Studies evaluating and describing the effectiveness of intervention strategies are needed. Specifically, comparative studies including multiple treatment options or patient populations would be useful.

## 5. Conclusions

The purpose of this review was to describe the evidence related to the injury characteristics and management strategies for circus artists. This review found injury trends and characteristics are well-researched but variably reported. Acrobats, particularly those incorporating ground elements, are most often injured, with shoulder, ankle, and spine injuries commonly reported. Although some risk factors are known and can predict who may develop injury, few sport-specific screening tools are available or used for this unique population. Further proactive assessment of psychological variables and screening instruments for this population appear warranted. Importantly, the physical therapy management of circus injuries is limited to few studies of low-level evidence and should be a point of emphasis in this scholarly area. While a growing population globally, the evidence related to physical therapy evaluation and management of circus artists would benefit from consistent reporting and high-quality prospective and comparative studies.

## Figures and Tables

**Table 1:** Studies reporting on injury patterns in circus artists.

Study	Study Design	Sample Size	Participant Characteristics	Mechanism of Injury	Injury Characteristics
Al-Kashmiri [29]	Case report	n = 1	26 y/o male	Overuse	Fibular stress fracture
Asselin [27]	Case series	n = 9	8 performers, 1 staff member Age range: 19–34 years 8 females, 1 male	Traumatic; fall while performing hair hanging during a ‘human chandelier’ maneuver	Fractures of the face, cervical, thoracic and lumbar spine, humerus, radius, wrist and hand, ribs, sacrum, femur, tibia, fibula, and ankle; concussions; lacerations of the knee, liver and scalp; pulmonary contusion; pneumothorax
Greenspan [17]	Prospective cohort	n = 21	Adolescents: Mean age: 14.7 (1.3) years 12 females Mean circus experience: 5.1 years Primary discipline: 35.9% aerial, 21.4% ground, 42.9% mixed Adults: Mean age: 30.7 (3.1) years 5 females, 4 males Mean circus experience: 10.5 years Primary discipline: 30% aerial, 40% ground, 20% mixed, 10% Chinese pole	Overuse (44% of adolescent injuries, 55% of adult injuries); Traumatic (56% of adolescent injuries, 45% of adult injuries)	Tissues involved (most to least frequent): joint, muscle/tendon, nerve, bone, central nervous system, integument. Body region involved (reported as lower extremity, upper extremity, spine): adolescents—44%, 33%, 22%; adults—32%, 45%, 24% Injury rate per 1000 session exposures was 3 for adolescents and 13 for adults Time-loss injury rates were 1.3 and 7.2, respectively Time-loss injury frequency: 44% adolescents, 55% adults
Greenspan [10]	Prospective cohort	n = 201	Mean age: 31.4 (8.9) years 16 adolescents, 185 adults 172 females, 29 males Mean circus experience: 7.0 (4.2) years 130 pre-professionals, 71 professionals	Acute–sudden (32%), repetitive–sudden (12%), repetitive–gradual (54%), unknown (1%)	Most common tissue involved: muscle/tendon injuries (52%) Most common body region involved: shoulder 22%, lumbosacral region 13%, elbow 10%, all others < 10% Injury rates per 1000 sessions: 3.8 overall, 2.42 adolescents, 4.0 adults, 3.5 female, 5.7 male, 5.9 aerialists with ground elements, 4.3 aerial, 3.7 ground (balance and control)
Greenspan [2]	Cohort	n = 24	Mean age: 21.4 (8.3) years 20 females, 4 males Mean circus experience: 7.3 (4.4) years	Overuse: 55.3% Traumatic: 44.7%	Most common tissue involved: joint (46.8%), muscle/tendon (25.5%), nerve (14.9%) all others < 10% Most common body region involved: shoulder 21.3%, wrist/hand 17.0%, hip/thigh and ankle/foot 12.8% each, upper trunk 10.6%, all others < 10% Injury rate per 1000 session exposures was 5 overall, 2.3 for aerialists, 7.0 for the ground group, 3.8 for the mixed group Time-loss injury frequency: 53.2%
Hakim [18]	Cohort	n = 31	Mean age: 22.5 (2) years 14 females, 17 males	Not specified	Most common body region involved: lower limb (44%; ankle 68%, knee 27%, tibia 5%), upper limb (32%; 50% shoulder, 25% wrist, elbow 19%, forearm 6%), trunk (16%), neck (8%)
Hamilton [31]	Secondary analysis of prospective injury data	n = 1281	Circus artists	Not specified	64% incurred at least 2 medical attention injuries 23.1% incurred at least 2 time-loss injuries
Kraan [16]	Systematic review	n = 67, 1 trapeze artist	Mean age of total sample: 27.1 years Male trapeze artist age not specified	Overuse	Posterior circumflex humeral artery pathology
Long [22]	Cross- sectional	n = 30	Mean age: 16.6 years	Not specified	13.3% reported losing at least one day of participation to injury. Fear of losing playing/practice time cited as most common reason to not report injury (42.9%)
Munro [19]	Prospective cohort	n = 63	Mean age: 22 years 33 females, 30 males	Not specified	Most common body region involved: spine 35%, ankle 26%, shoulder 12%, all others < 10% Injury activity frequency: acrobatics/tumbling (23%), handstands (12%), adagio (11%), Chinese pole (10%), others < 10%
Orlando [20]	Cohort	n = 584	208 females, 378 males Mean age: females, 25.3 (9.4) years; males: 30.3 (9.4) Type of performer: females—sudden load (71.4%), non-sudden load (16.5%), musician (12.1%); males—sudden load (66.7%), non-sudden load (18.0%), musician (15.3%)	Not specified	Rate ratio estimates for medical attention injuries = 1.5, TL-1 = 1.3, TL-15 = 1.1
Rossini [23]	Cross-sectional	n = 31	Mean age: 21.1 (2.6) years 18 females, 13 males Mean circus experience: 8.0 (4.3) years	Not specified	Lumbar multifidi asymmetry in prone was greater in artists reporting LBP in the past 4 weeks or 3 months
Russell [28]	Narrative review	Not applicable	Dancers, circus performs, theatre performers, and film/television performers	Traumatic	Notes concussions may be classified under ‘head and neck’ category of injury reporting
Shrier [25]	Descriptive epidemiological	n = 962	Professional artists Mean age: not reported 303 females, 659 males	Not specified	89.4% incurred an injury requiring medical attention, 74.2% incurred at least 1 TL-1 injury, 50.8% incurred at least 1 TL-15 injury Injury risk per 1000 performances: 5.1 medical attention, 2.5 TL-1, 0.86 TL-15
Shrier [24]	Descriptive epidemiological	n = 1376	Professional artists Mean age: not reported 534 females, 842 males Primary discipline: 80.5% acrobats, 11.8% non-acrobats, 7.8% musicians	Not specified	Types of injury (most to least frequent): muscle/tendon, other, joint and ligament, contusions/lacerations, fractures and bone, nerve Body region involved (most to least frequent): lower extremity, upper extremity, spine, head and neck, trunk, other Injury rates per 1000 performances: overall 9.7, acrobats 11.2, non-acrobats 6.8, musicians: 4.3 4.4% injuries classified as TL-15
Stubbe [4]	Prospective cohort	n = 44	Mean age: 22.0 (2.8) years 21 females, 23 males	Not specified	Most common body region involved: shoulder 27.7%, lower back 15.8%, wrist 14.1%, all others < 10% Mean duration of injury: 6.9 days Injury incidence rate of 3.3 injuries/1000 h
Stuckey [21]	Longitudinal	n = 334	Mean age: 19.2 (2.1) years 134 females, 200 males	Not specified	Overall injury rate per 1000 training hours = 1.89; rate of injuries lasting more than 4 weeks per 1000 training hours = 0.94 Clinical incidence of injury/student/year = 1.78, which decreased over time with experience
Wanke [26]	Retrospective descriptive epidemiologic	n = 169	Age range: 11–22 years 99 females, 70 males	Traumatic	Most common tissue involved: joint (48.8%), bone (23.8%), ligament (14.9%) all others < 10% Most common body region involved: lower extremity (37%), cervical spine (28.3%), upper extremities (19.6%), thoracic spine (17.2%), lumbar spine (10.9%) Injury risk per 1000 h = 0.3
Wojciuk [30]	Case report	n = 1	64 y/o female 8-year history of an acrobat	Overuse	Bilateral upper limb arterial stenosis
Wolfenden [7]	Systematic review	n = 4795, 8 studies included	High school through professional circus artists	Floor acrobatics/tumbling most common mechanism (23–50.3%) followed by handstands (12%), adagio (11%) and Chinese pole (10%)	Most common type of injury: soft tissue Most common body region involved: spine and ankle Injury rate per 1000 exposures ranged from 7.4 to 9.3

**Table 2:** Risk factors for circus artist injuries.

Study	Study Design	Sample Size	Participant Characteristics	Reported Risk Characteristics
Bolling [32]	Qualitative	n = 82	Professional coaches, physiotherapists, athletic trainers and performance medicine therapists, and artists Participant demographics not reported	Injury risk reported to increase with higher workload. Busy daily schedule, more cues, fewer rotations and backups, less rest/recovery time, higher mental load, fatigue, improper technique, and external factors (weather, area, and touring conditions) increased risk
Faltus [33]	Narrative review	Not applicable	Circus artists	Risk increases with higher volume and frequency of circus performances, lack of a true “off season”, certain cultural considerations (e.g., language barriers and pain ideologies), certain psychological considerations (e.g., emotional, social and cognitive loads)
Greenspan [17]	Prospective cohort	n = 21	Adolescents: Mean age: 14.7 (1.3) years 12 females Mean circus experience: 5.1 years Primary discipline: 35.9% aerial, 21.4% ground, 42.9% mixed Adults: Mean age: 30.7 (3.1) years 5 females, 4 males Mean circus experience: 10.5 years Primary discipline: 30% aerial, 40% ground, 20% mixed, 10% Chinese pole	Higher age predictive of injury risk
Greenspan [10]	Prospective cohort	n = 201	Mean age: 31.4 (8.9) years 16 adolescents, 185 adults 172 females, 29 males Mean circus experience: 7.0 (4.2) years 130 pre-professionals, 71 professionals	Extrinsic risk factors: Discipline (aerial with ground elements), pre-professional (less experience) Intrinsic risk factors: Age (adults more than adolescents), female sex, disordered eating history
Hamilton [34]	Secondary analysis	n = 1281	Circus artists	Injury risk dependent on injury definition Sudden-load artists and age > 20 years had increased risk across injury classifications. Female sex and age > 30 years had increased risk for medical attention injuries. European artists more likely to sustain time-loss injuries than North American artists.
Hamilton [31]	Secondary analysis	n = 1281	Circus artists	Previous injury not identified to be a casual risk factor.
Orlando [20]	Cohort	n = 584	208 females, 378 males Mean age: females, 25.3 (9.4) years; males: 30.3 (9.4) Type of performer: females—sudden load (71.4%), non-sudden load (16.5%), musician (12.1%); males—sudden load (66.7%), non-sudden load (18.0%), musician (15.3%)	Injury risk is higher among sudden load and non-sudden load artists for medical attention and time loss injuries after breaks (2 days post activity)
Russell [28]	Narrative review	Not applicable	Dancers, circus artists, theatre, film and television performers	Suggests while circus artists sustain concussions, determining risk is limited by barriers in reporting and injury pattern definition.
Shrier [9]	Retrospective cohort	n = 47	Professional circus artists 17 females, 30 males	Prior injury, emotional exhaustion, self-efficacy, and fatigue were associated with an increased in injury risk. Conflicts or pressure not associated with increased injury risk. Low self-efficacy had the strongest relationship.
Shrier [25]	Descriptive epidemiological	n = 962	Professional artists Mean age: not reported 303 females, 659 males	Observed injury risk was not consistent with theoretical injury risk in circus performers.

**Table 3:** Studies reporting on screening tools or principles in circus artists.

Study	Study Design	Sample Size	Participant Characteristics	Instrument/ Process Description	Outcome(s)
Burnstein [35]	Cohort	n = 238	Professional circus performers 76 females, 162 males Mean age: 28.7 (6.4) years	Physical capacity assessment which included dynamic balance, modified Harvard step test, grip strength, vertical jump test, pull-up test, and 60 s jump test; data collected at baseline, 6, 12, and 18 months	Acceptable test–retest reliability over long periods of time for handgrip, vertical jump, and pull-up assessments; Harvard step test and 60 s jump test had poor reliability with comparison of baseline but acceptable reliability otherwise; Dynamic balance test never reached a level of acceptability at any time point; unclear if same tester was used
Callahan [36]	Narrative review	Not applicable	Dancers and circus artists	Suggested subjective screening: artist identity and participation level, injury management, habitual postures, fatigue, sleep, nutrition, menstrual history, support systems, other activities and participation; Suggested physical screening: The Beighton Score, Brighton criteria, 5-point screening questionnaire, The Lower Limb Assessment Score, The Upper Limb Hypermobility Assessment Tool, assessment of strength, functional movements, closed and open chain activities	Not applicable
Decker [37]	Longitudinal	n = 92	Circus students 32 females, 60 males Mean age: 20.9 (2.4) years	Modified Consensus Sleep Diary, ratings of fatigue, wakefulness and perceived exertion; measures taken for 7 consecutive days at 4 different time points	Significant fluctuations in fatigue, wakefulness, and perceived exertion over time points with increased challenge to achieve adequate sleep noted at end of year. Significant correlation between wakefulness and other sleep parameters (duration, latency, and quality). Sleep quality and perceived exertion related to fatigue. No significant differences between disciplines, sex, or year in program.
Donohue [38]	Cross-sectional	n = 109	Professional circus artists (n = 88) and circus students (n = 21) 42 females, 67 males Mean age: 28.5 (7.3) years	Sport Interference Checklist Student Athlete Relationship Instrument; Patient-Reported Outcomes Measurement Information System (anxiety, depression, satisfaction with social roles and activities, social isolation, emotional support, informational support, fatigue, sleep disturbance); Social Skill in Work Environment; satisfaction with overall circus performance	Mental health: Students scored higher for anxiety and professionals demonstrated lower levels of depression. Social health: Professionals scored higher on informational and emotional support and social skills, and lower on social isolation than students. Physical health: Students reported higher fatigue. Performance: Professionals reported less interferences than students. Professionals reported higher overall satisfaction with performance, social roles and responsibilities than students.
Greenspan [39]	Cross-sectional	n = 201	Professional (n = 71) and pre-professional (n = 130) 172 females, 29 males Age range: 13–69 years	Physical examination battery of upper and lower extremity flexibility, strength, and balance and cardiorespiratory measures to determine normative data	Mobility: Females had more lumbar mobility, shoulder and hip (except for external rotation) passive range of motion, hamstring and straddle flexibility, males had more pectoralis minor flexibility; Younger participants generally had more mobility than older counterparts; Strength: Males demonstrated more upper body strength, females had greater hip abduction strength; Professionals demonstrated more lower abdominal and upper body strength; Professionals had lower recovery heart rates than pre-professionals; Findings were not discipline-specific.
Huberman [40]	Cross-sectional	n = 189	Professional and amateur acrobats 157 females, 30 males Mean age = 31.9 years Acrobatic subgroup: aerial (n = 40), ground (n = 21), both (n = 128)	Shoulder range of motion and strength, grip strength	Males and females demonstrated significantly more shoulder range of motion, except for flexion, than established norms Acrobats had greater shoulder strength than the general population, while males had significantly less grip strength than the general population. No significant strength or range of motion differences across age groups.
Rossini [23]	Cross-sectional	n = 33	Circus artist students 19 females, 14 males Mean age: 21.2 (2.5) years Discipline: floor acrobatics (n = 11), aerial acrobatics (n = 15), balancing (n = 6), juggling (n = 1)	Self-reported training, injury and low back pain history; Oswestry Disability Index, Athlete Disability Index, Pain Catastrophizing Scale	Positive correlation between athlete and Oswestry disability indices. Athlete disability index positively correlated with pain intensity and pain catastrophizing. No significant correlations between pain duration and assessment tools.
Scherb [41]	Clinical suggestion	Not applicable	Circus artists with low back pain	Movement system impairment classification	Suggestion based on expert opinion to match the movement needs of circus artists
Shrier [42]	Prospective longitudinal	n = 36	Professional circus artists 16 females, 21 males Median age = 32.4	State-Trait Anxiety Inventory, Ways of Coping, Profile of Mood States, Likert scales to assess anxiety, sleep, confidence, fatigue, well-being; assessed at baseline, daily, weekly for 4 weeks	Monitoring psychological states were generally successful regarding time to complete, distribution, acceptability, and comprehension of questionnaires. Respondents believed questionnaires could be burdensome to complete at expected rate.
Smith [43]	Cross-sectional	n = 15	Pre-professional circus artists 15 females Mean age: 13.6 (2.3) years	Isokinetic testing of knee and ankle, trunk strength, lumbar range of motion, lower extremity flexibility; Modified SafeDance IV survey report	Acrobats generally demonstrated more than normal range of motion. Significant peak torque correlations noted between the ankle and hip/knee. Although the ankle was most commonly injured, and often hypermobile, most injuries were not related to acrobatic participation.

**Table 4:** Studies related to the physical therapy management of circus artists.

Study	Study Design	Sample Size	Participant Characteristics	Intervention	Frequency/Duration	Comparison	Outcome(s)
Callahan [36]	Narrative review	Not applicable	Hypermobility in aesthetic performing artists	Suggested patient-specific utilization of interventions such as bracing, education (self-management, pain science, fatigue), posture and movement retraining, strength and proprioceptive training	Not applicable	Not applicable	Not applicable
Chimenti [44]	Case report	n = 1	16 y/o female Clinical diagnosis: lumbar extension-rotation syndrome	1. Education regarding movement impairments 2. Home exercises and taping to minimize lumbar extension–rotation/improve abdominal and gluteal recruitment and hip flexor flexibility	16 visits over 16 weeks	Not applicable	Assessed at baseline, at each phase of intervention, and discharge; Clinically significant improvement in pain and function
Faltus [33]	Narrative review	Not applicable	Circus performance artist and acrobat	Suggested use of trans-disciplinary approach for management of the population	Not applicable	Not applicable	Not applicable
Fecteau [45]	Case report	n = 1	15 y/o female Clinical diagnosis: transitional vertebrae and lumbar retrolisthesis	1. Manual therapy for lumbar hypomobility 2. Therapeutic exercise for hip strengthening 3. Neuromuscular re-education for lumbopelvic stability 4. Sport-specific training	16 visits over 8 weeks	Not applicable	Assessed at baseline, 5th and 10th visit; Clinically significant improvement in pain; No functional measure or self-reported questionnaire reported
Ganderton [46]	Cohort	n = 29	13 females, 16 males Active touring professional circus performers	Feasibility study for calf muscle training Maximal set of calf raises with proper form, each limb, once daily each workday	47 exercise sessions over 9 weeks	Not applicable	Feasibility and endurance assessed at study completion; injury questionnaire administered weekly; High adherence and substantial improvement noted in calf endurance
Ganderton [47]	Cohort	n = 24	Mean age: 23.82 (2.88) 16 females, 8 males, student circus performers Clinical diagnosis: atraumatic shoulder instability	Telehealth delivery mode Phasic exercise progression emphasizing scapular control and strengthening in increasing ranges of motion towards circus specific training	12 visits over 12 weeks	Not applicable	Assessed at baseline, 6 and 12 weeks, 6 and 9 months; Statistically and clinically significant improvement in function, strength and kinesiophobia
Roubea [48]	Case report	n = 1	16 y/o female acrobat Clinical diagnosis: partial tear right UCL	Phasic progression consisting primarily of 1. Manual therapy for pain relief 2. Progressive wrist, elbow and scapular strengthening, core stability progressing to closed chain activities, plyometric and sport-specific exercise	10 visits over 8 weeks	Not applicable Not applicable	Assessed at baseline, weeks 3, 6, 10 and 3 months; Clinically significant improvements in pain and function
Scherb [41]	Clinical suggestion	Not applicable	Not applicable	Suggestion to use movement system impairment classification in management of circus athletes	Not applicable	Not applicable	Not applicable
Teo [49]	Case report	n = 1	42 y/o male rigger Clinical diagnosis: flexor carpi ulnaris tear	1. Wrist splinting × 6 weeks 2. Intermittent mobilization at 4 weeks 3. Progressive forearm strengthening and loading activities at 6 weeks	Not reported	Not applicable	Return to normal activity at 3 months, pain and grip assessed at 6 months; Clinically significant improvement in pain and grip strength

**Table 5:** Studies reporting on psychosocial variables in circus artists.

Study	Study Design	Sample Size	Participant Characteristics	Instrument(s) Used	Outcomes
Cayrol [5]	Qualitative	n = 10	Professional circus artists 5 females, 5 males Mean age: 33 (range 27–42) years Mean performance experience: 12 years At least one injury reported	Semi-structured, individual interviews (virtual or in-person)	4 themes of perceptions and beliefs of injury identified: 1. The injured artists—circus training and performance viewed as their whole life, not a job; injury has negative effect on mood and emotion. 2. Professionalism—work and the performance should take precedence over personal issues or injuries. 3. Circus life—work perceived as challenging, lack of a fixed address limited injury management, injuries often self-managed or treated by other artists. 4. Artists’ experience of healthcare—personalized and specialized healthcare is required but not typically available.
Decker [37]	Prospective cohort	n = 92	Professional circus students 32 females, 60 males Mean age: 20.4 (2.4) years	Circus Daily Challenges Questionnaire; Scales perceiving coping, state anxiety, sleep and distress	Highest scores of challenge level, state anxiety, and fatigue at end of year Schedule demands overall highest challenge item. Technical development, artistic expression, physical prep, and sleep 2nd highest challenge item. Sleep, artistic expression, and fear of injury were most frequently reported over the year. Increase in challenge level was associated with an increase in anxiety state and fatigue.
Shrier [9]	Retrospective cohort	n = 47	Professional circus artists 17 females, 30 males	The Recovery-Stress Questionnaire	High measures of fatigue, emotional exhaustion, injury, and low self-efficacy were associated with a 2–3× increase in risk for injury. High levels of social stress or a low level of either success or personal accomplishment may be a predictor of injury.
Stubbe [51]	Prospective cohort	n = 98 total; circus artists n = 25	Performing arts students 74 females, 24 males Mean age: 19.9 (2) years	Oslo Sports Trauma Research Centre Questionnaire; Stress (0–100, 100 = more stressed); Sleep quality (1–10, 10 = good sleep); Subjective mental health (higher score = worse), loneliness and mental health Inventory	Higher prevalence of mental health problems during 3-month COVID-19 lockdown. Average stress 40.38 pre-COVID-19 and 37.66 during lockdown. Average sleep quality 6.44 sleep quality pre-COVID, 6.87 during lockdown. Subjective mental health: 27.6% in March, 32.7% in April, 35.7% in May. Loneliness: 75.5% of people dealt with severe loneliness during lockdown.
van Rens [52]	Explanatory research	n = 248	Expertise included amateurs, students, professionals, retired professionals 188 females, 54 males, 6 other/prefer not to say Mean age: 30.7 (8.4) years Mean circus experience: 7.3 (6.7) years	Sensation seeking, Emotional regulation, and Agency Scale; Ten Item Personality Inventory; Accidents and Close Calls in Sport Inventory; Perceived risk of Activities	No significant difference between circus categories for sensation. Aerial acrobats experienced more emotional regulation, agency, conscientiousness and agreeableness than object manipulators. Floor acrobats reported more near misses and accidents than both aerialists and object manipulators. Object manipulators had significantly lower perceived risk than aerial and floor acrobatics.
van Rens [11]	Qualitative	n = 8	Professional circus artists who were formerly gymnasts 4 female, 4 male Mean age: 30.9 (4.4) years Mean circus experience: 5.8 years	Semi-structured individual interview	3 phases were identified in transitioning from gymnastics to professional circus: (1) realizing, (2) adaptation, (3) thriving. Realizing phase included hard work, motivation to accomplish the transition, social support, optimism. Adaptation phase included general stress, loss of competence, social adjustment, taking calculated risks, physical recovery. Thriving phase included freedom, personal development, social connectedness. Career transition process included an emergence of a circus artist identity.
van Rens [8]	Explanatory research	n = 500	Expertise included amateurs, students, professionals, retired professionals 415 females, 62 males, 23 transgender/gender-diverse Mean age: 31.1 (8.2) years Mean circus experience: 7.6 (5.9) years	Depression Anxiety Stress Scale-21; The Flourishing Scale; State Trait Assessment of Resilience	Circus artists experience lower levels of mental health than other populations despite higher levels of psychological resilience. Circus artists had higher levels of depression, anxiety, and stress, and lower flourishing than other populations, with object manipulators reporting worse scores than other disciplines. Higher state/trait resilience associated with higher psychological well-being. Transgender and gender-diverse individuals had higher depression scores.
van Rens [53]	Explanatory research	n = 500	Expertise included amateurs, students, professionals, retired professionals 415 females, 62 males, 23 transgender/gender-diverse Mean age: 31.1 (8.2) years Mean circus experience: 7.6 (5.9) years	Circus Addiction Inventory; Eating Attitudes Test short form; State Trait Assessment of Resilience	Exercise addiction positively related to being at risk of eating disorders and negatively related to experience. Males scored lower on the circus addiction inventory than female or transgender/gender-diverse peers. Aerial acrobatics were more likely to be at risk for eating disorders than floor artists and had higher addiction scores compared to equilibrium and floor acrobatics.
Walby [54]	Qualitative	n = 31	Circus aerialists 19 females, 12 males Age range: 22–37 years	Semi-structured individual interview	Primary emergent themes included training with pain, body image, performance, aerialism and risk, injury and the erosion of body capital, and aging out. Recognizing the circus performance span is brief, artists often push their bodies through pain and risk boundaries.

## Data Availability

All relevant data are provided within the manuscript and in the Supplementary Materials.

## Supplementary Materials

The following supporting information can be downloaded at https://www.mdpi.com/article/10.3390/jcm14175948/s1: Supplementary: Circus artist scoping review search strings.
