# Supplementary material for: Injury Characteristics and Physical Therapy Management Strategies for Circus Artists: A Scoping Review
Source: J Clin Med. 2025 Aug 22;14(17):5948. doi: 10.3390/jcm14175948 (PMC12429121; doi:10.3390/jcm14175948)
Supplement: Supplementary file 1 [file jcm-14-05948-s001.zip › jcm-3788158-supplementary.pdf]

Supplementary. Circus artist scoping review search strings.

General Search String:

("circus art*"[Title/Abstract] OR "circus"[Title/Abstract] OR "aerialist*"[Title/Abstract] OR "acrobat*"[Title/Abstract] OR "trapeze"[Title/Abstract]) AND ("wounds and injuries"[MeSH Terms] OR "epidemiologic methods"[MeSH Terms] OR "risk factors"[MeSH Terms] OR "psychiatry and psychology category"[MeSH Terms] OR "physical therapy modalities"[MeSH Terms] OR ("wound*"[Title/Abstract] OR "injur*"[Title/Abstract] OR "epidemiologic methods"[Title/Abstract] OR "risk factors"[Title/Abstract] OR "depress*"[Title/Abstract] OR "anxiety"[Title/Abstract] OR "fear"[Title/Abstract] OR "kinesiophobia"[Title/Abstract] OR "stress"[Title/Abstract] OR "physical therapy modalities"[Title/Abstract] OR "physical therapy"[Title/Abstract])) NOT ("Animals"[Mesh] NOT ("Animals"[Mesh] AND "Humans"[Mesh]))

PubMed:

("circus art*"[tiab] OR circus[tiab] OR aerialist*[tiab] OR acrobat*[tiab] OR trapeze[tiab]) AND ("wounds and injuries"[Mesh] OR "epidemiologic methods"[Mesh] OR "risk factors"[Mesh] OR "psychiatry and psychology category"[Mesh] OR "physical therapy modalities"[Mesh] OR (wound*[tiab] OR injur*[tiab] OR "epidemiologic methods"[tiab] OR "risk factors"[tiab] OR depress*[tiab] OR anxiety[tiab] OR fear[tiab] OR kinesiophobia[tiab] OR stress[tiab] OR "physical therapy modalities"[tiab] OR "physical therapy"[tiab])) NOT (Animals[Mesh] NOT (Animals[Mesh] AND Humans[Mesh]))

CINAHL:

((TI "circus art*" OR AB "circus art*") OR (TI circus OR AB circus) OR (TI aerialist* OR AB aerialist*) OR (TI acrobat* OR AB acrobat*) OR (TI trapeze OR AB trapeze)) AND ((MH "wounds and injuries+") OR (MH "epidemiologic methods+") OR (MH "risk factors+") OR (MH "psychiatry and psychology category+") OR (MH "physical therapy modalities+") OR ((TI wound* OR AB wound*) OR (TI injur* OR AB injur*) OR (TI "epidemiologic methods" OR AB "epidemiologic methods") OR (TI "risk factors" OR AB "risk factors") OR (TI depress* OR AB depress*) OR (TI anxiety OR AB anxiety) OR (TI fear OR AB fear) OR (TI kinesiophobia OR AB kinesiophobia) OR (TI stress OR AB stress) OR (TI "physical therapy modalities" OR AB "physical therapy modalities") OR (TI "physical therapy" OR AB "physical therapy"))) NOT ((MH Animals+) NOT ((MH Animals+) AND (MH Humans+)))

SPORTDiscus:

((TI "circus art*" OR AB "circus art*") OR (TI "circus" OR AB "circus") OR (TI "aerialist*" OR AB "aerialist*") OR (TI "acrobat*" OR AB "acrobat*") OR (TI "trapeze" OR AB "trapeze")) AND (DE "wounds and injuries" OR DE "epidemiologic methods" OR DE "risk factors" OR DE "psychiatry and psychology category" OR DE "physical therapy modalities" OR ((TI "wound*" OR AB "wound*") OR (TI "injur*" OR AB "injur*") OR (TI "epidemiologic methods" OR AB "epidemiologic methods") OR (TI "risk factors" OR AB "risk factors") OR (TI "depress*" OR AB "depress*") OR (TI "anxiety" OR AB "anxiety") OR (TI "fear" OR AB "fear") OR (TI "kinesiophobia" OR AB "kinesiophobia") OR (TI "stress" OR AB "stress") OR (TI "physical therapy modalities" OR AB "physical therapy modalities") OR (TI "physical therapy" OR AB "physical therapy"))) NOT (DE "Animals" NOT (DE "Animals" AND DE "Humans"))

Scopus:

(TITLE-ABS("circus art*") OR TITLE-ABS(circus) OR TITLE-ABS(aerialist*) OR TITLE-ABS(acrobat*) OR TITLE-ABS(trapeze)) AND (INDEXTERMS("wounds and injuries") OR INDEXTERMS("epidemiologic methods") OR INDEXTERMS("risk factors") OR INDEXTERMS("psychiatry and psychology category") OR INDEXTERMS("physical therapy modalities") OR TITLE-ABS(wound*) OR TITLE-ABS(injur*) OR TITLE-ABS("epidemiologic methods") OR TITLE-ABS("risk factors") OR TITLE-ABS(depress*) OR TITLE-ABS(anxiety) OR TITLE-ABS(fear) OR TITLE-ABS(kinesiophobia) OR TITLE-ABS(stress) OR TITLE-ABS("physical therapy modalities") OR TITLE-ABS("physical therapy")) AND NOT ((INDEXTERMS(animals OR animal)) AND NOT (INDEXTERMS(humans OR human)))

Cochrane:

(("circus" NEXT art*):ti,ab OR circus:ti,ab OR aerialist*:ti,ab OR acrobat*:ti,ab OR trapeze:ti,ab) AND ([mh "wounds and injuries"] OR [mh "epidemiologic methods"] OR [mh "risk factors"] OR [mh "psychiatry and psychology category"] OR [mh "physical therapy modalities"] OR (wound*:ti,ab OR injur*:ti,ab OR "epidemiologic methods":ti,ab OR "risk factors":ti,ab OR depress*:ti,ab OR anxiety:ti,ab OR fear:ti,ab OR kinesiophobia:ti,ab OR stress:ti,ab OR "physical therapy modalities":ti,ab OR "physical therapy":ti,ab)) NOT ([mh Animals] NOT ([mh Animals] AND [mh Humans]))

Google Scholar:

("circus artist*" OR circus OR aerialist* OR acrobat* OR trapeze) AND (("Injur*" OR "Epidemiologic Methods" OR incidence OR prevalence OR "Risk Factors") OR (injury AND (prevention OR assessment OR screening)) OR (depression OR anxiety OR stress OR

psychosocial) OR ("Physical Therapy Modalities"[Mesh] OR "physical therapy" OR physiotherapy OR rehabilitation OR recovery OR "conservative management"))
